# Hybrid Polymer Composites Used in the Arms Industry: A Review

**DOI:** 10.3390/ma14113047

**Published:** 2021-06-03

**Authors:** Kamil Czech, Rafał Oliwa, Dariusz Krajewski, Katarzyna Bulanda, Mariusz Oleksy, Grzegorz Budzik, Aleksander Mazurkow

**Affiliations:** 1 Doctoral School of Engineering and Technical Sciences at the Rzeszow University of Technology, 35-959 Rzeszow, Poland; d516@stud.prz.edu.pl; 2 Department of Polymer Composites, Faculty of Chemistry, Rzeszow University of Technology, 35-959 Rzeszow, Poland; d.krajewski@prz.edu.pl (D.K.); k.bulanda@prz.edu.pl (K.B.); molek@prz.edu.pl (M.O.); 3 Department of Machine Construction, Faculty of Mechanical Engineering and Aeronautics, Rzeszow University of Technology, 35-959 Rzeszow, Poland; gbudzik@prz.edu.pl (G.B.); almaz@prz.edu.pl (A.M.)

**Keywords:** polymer composites, shear thickening fluid, nanofillers, fiber, ballistic properties

## Abstract

Polymer fiber composites are increasingly being used in many industries, including the defense industry. However, for protective applications, in addition to high specific strength and stiffness, polymer composites are also required to have a high energy absorption capacity. To improve the performance of fiber-reinforced composites, many researchers have modified them using multiple methods, such as the introduction of nanofillers into the polymer matrix, the modification of fibers with nanofillers, the impregnation of fabrics using a shear thickening fluid (STF) or a shear thickening gel (STG), or a combination of these techniques. In addition, the physical structures of composites have been modified through reinforcement hybridization; the appropriate design of roving, weave, and cross-orientation of fabric layers; and the development of 3D structures. This review focuses on the effects of modifying composites on their impact energy absorption capacity and other mechanical properties. It highlights the technologies used and their effectiveness for the three main fiber types: glass, carbon, and aramid. In addition, basic design considerations related to fabric selection and orientation are indicated. Evaluation of the literature data showed that the highest energy absorption capacities are obtained by using an STF or STG and an appropriate fiber reinforcement structure, while modifications using nanomaterials allow other strength parameters to be improved, such as flexural strength, tensile strength, or shear strength.

## 1. Introduction

Over the years, composite materials, especially polymeric fiber composites, have gained popularity in every industry sector. The high mechanical and thermal strength, low specific gravity, and weather resistance make these composites a competitive construction material compared to traditional materials, such as wood, steel, and concrete. Composites are used extensively in the construction, aerospace, automotive, and sports equipment sectors. The physical and mechanical properties of polymer composites are closely related to the type and modification of the polymer matrix, the structure and composition of the reinforcement, and the constituent elements. A classical composite material is composed of a matrix-coated reinforcement. The polymer matrix can be a thermoplastic polymer (polycarbonate, polyamide) or a duroplastic resin (epoxy, polyester). The composite reinforcement can be in the form of fabrics, mats (glass, carbon, aramid, basalt, or hybrid fiber), or powder fillers dispersed in the matrix. The main function of the composite reinforcement is to carry external loads. The properties of the fibers forming the composite reinforcement play a key role here. They should have high tensile strength and Young’s modulus, low elongation at break, and low density. Such fibers are referred to as high-performance fibers, which include (Table 1) glass fibers of E and S type, carbon fibers, and ceramic and polymer fibers (p-aramids, high-molecular-weight UHMWPE-polyethylene, and aromatic polyesters). A common characteristic of these fibers is that their tensile strength and Young’s modulus increase with decreasing diameter, at the expense of decreasing elongation at break. Due to their properties, these fibers can find potential applications in the arms industry. However, the most popular and widely used fibers are glass, carbon, and aramid reinforcements [1,2,3,4].

**Figure 1:**
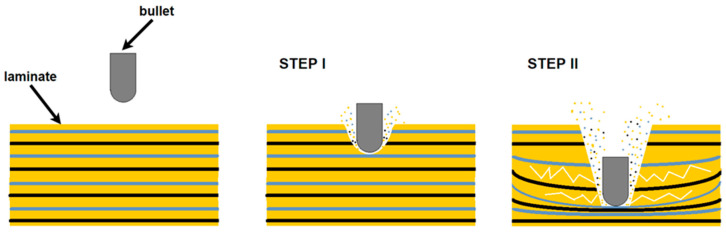
Two-stage laminate destruction process based on [13]. (From open access publication).

High-strength fibers are also preferred by the arms industry and have replaced steel structures, with composites reinforced mainly with aramid fabrics. Modern military conflicts are characterized by increasing asymmetry, i.e., a significant disproportion of equipment, weaponry, technology, and resources between the fighting sides. The weaker side usually adopts a strategy of offensive, partisan warfare. The attack-and-escape tactic is characterized by close-range combat, continuous movement of forces, surprise attacks, traps, and improvised explosive devices [9]. The experience of Russian troops in the fighting in Afghanistan and Chechnya shows the effectiveness of partisan tactics. During ambushes, heavily armored tanks and combat vehicles, due to their heavy weight, had difficulty performing maneuvers. They became easy targets for anti-tank weapons. At close range, the classical armor was no obstacle for an anti-tank missile. Asymmetric warfare forced the vehicle armor and the materials from which it was made to be modified. Until now, vehicles were reinforced with steel armor. To increase protection, the armor was thickened, significantly increasing its weight. However, this reduced mobility, increased fuel consumption, and made air transport impossible. The ideal solution was the use of polymer composites. Currently, polymer fiber composites are used by the arms industry to produce not only helmets and inserts for bulletproof vests but also ballistic shields for light armored vehicles, patrol boats, and helicopters. The biggest advantage of composites is their low weight in comparison to steel. This translates into a reduction in vehicle weight, while maintaining full mobility and the same level of crew protection [10]. Resins, including epoxy, are mainly used as the matrix due to their good mechanical and thermal properties. They are resistant to moisture and most chemicals (including oils and greases) and are characterized by low shrinkage after hardening and ease of processing. We can divide the composite armor into inner cladding and outer ballistic panels. The former is designed to catch metal pieces of the inner side of the vehicle hull that have broken off after missile impact [9]. A ballistic shield consists of an outer ceramic layer and a multilayer laminate underneath. The function of ceramic panels is to absorb the impact energy, reduce the velocity of and crush the projectile blade, and change the direction of penetration. The composite performs the role of the ceramic. An additional function is to completely break and catch the projectile or its fragments [11]. Penetration of the laminate by a projectile is a complex process involving two stages of destruction (Figure 1). First, the impact energy causes shearing of the facing matrix layers and reinforcement, leading to fiber breakage. Shear destruction absorbs most of the projectile’s energy, which is lost with the successive layers. Second, the matrix is destroyed and the fibers are stretched at the point of impact energy concentration, which leads to interfacial delamination [12,13].

In practice, layouts of fabric-reinforced epoxy laminates alone do not create an effective or efficient shield. Adding more layers of fabric increases the thickness and weight of the armor, which is not a good approach. Therefore, the first and most important stage of designing composite materials for the arms industry is appropriate selection of the matrix and the reinforcement; the fundamental requirement is that they must be as light as possible, be mechanically strong, and also be able to absorb large amounts of energy. Therefore, the arms industry is looking for new material and construction solutions, which is also a challenge for scientists [14,15,16,17,18,19,20]. Considering the design assumptions, the roles and tasks of composite materials in ballistic shields, and the required mechanism of action of structural materials used in the arms industry, the current work focuses on the development of hybrid composites (materials consisting of two or more types of matrixes or/and reinforcements) [21]. Hybrid fiber composites are obtained by modifying the matrix and reinforcing it by introducing nanofillers into it, grafting nanofillers on the surface of the fibers, impregnating fabrics using a shear thickening fluid (STF) or a shear thickening gel (STG), or using a combination of these techniques. In addition, the physical structures of the composites are modified by the hybridization of fibers; the appropriate design of roving, weaving, and mutual orientation of fabric layers; and the development of 3D structures.

The constant development of weapons and warfare agents and the numerous methods of modifying polymer composites in order to improve their performance in protective applications confirm that the topic of hybrid fiber composites dedicated to the arms industry is interesting for scientists and important in terms of application. However, there are no review articles focusing on the achievements in this field to date. Therefore, this article discusses the modifications used to improve the ability of composites to absorb energy and other mechanical properties. The composites are categorized based on three basic fabrics: glass, carbon, and aramid. The focus is on the technologies of the applied solutions, in particular matrix modification and reinforcement with nanofillers and STFs. Their effects on the properties of composites are analyzed, and basic knowledge of the design assumptions related to the selection of fabrics and their orientation is assessed.

Based on the review, the best possibilities for energy absorption are revealed by using an STF or STG and an appropriate structure of the fibrous reinforcement. Modification with nanomaterials allows for the improvement of other strength parameters. Unfortunately, a significant part of the literature does not contain information about the mass of the developed hybrid composites, which is important as it largely determines the application possibilities of the discussed modification methods.

## 2. Hybrid Composites

In recent years, many scientific publications, including those on composites used in the arms industry, have been devoted to hybrid polymer composites. The main reason for developing hybrid polymer composites is the continuous search for new materials that, in addition to a favorable weight, are characterized by improved functional properties, including impact strength and durability. A well-designed hybrid composite uses the advantages of its individual components to minimize the disadvantages resulting from individual use of those components [22,23].

The continuous development of hybrid composite materials is associated with the search for new modifiers and nanofillers with unique functional properties, whose small contribution to the composite significantly improves its properties. Moreover, in polymeric fiber composites, nanofillers play an important role: when added separately or in several combinations, they improve the morphology of the composites, which, in turn, translates into improvement in their functional properties. The structure of the reinforcement is also important in terms of the weave and fiber structure, the arrangement of reinforcement layers in relation to each other at different angles, the use of different fibers, and the use of appropriate surface preparation of the reinforcing material [3,4,23].

### 2.1. Composites with the Addition of Nanofillers

**Figure 2:**
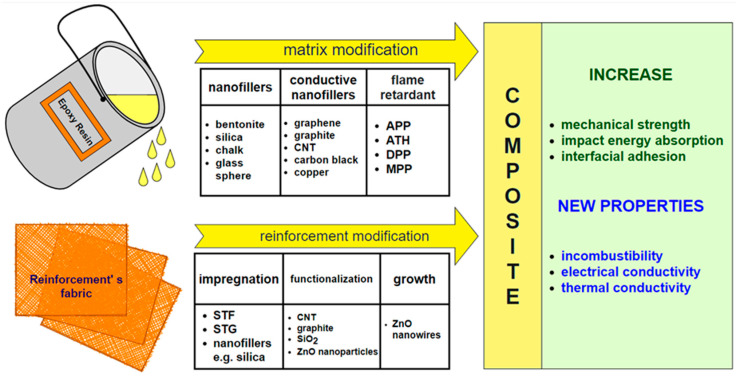
Summary effect of individual matrix modifications and fibrous reinforcement on the performance of epoxy composites.

There are many publications on the preparation of polymer nanocomposites in which nanofiller particles are uniformly distributed and one of the dimensions of these particles does not exceed the nano size. In addition to fibrous materials, nanoparticles in the form of plates, spheres, tubes, or rods can also be used as reinforcement in these composites. These include inorganic nanofillers, such as bentonite, silica, and metals (copper, zinc, silver, etc.) and their oxides, as well as organic ones, such as carbon black, graphene, graphite, carbon nanotubes (CNTs), and polymethyl methacrylate (PMMA) (Figure 2) [24].

Compared to fiber composites, composites reinforced with hybrid nanoparticles and fibers show improved mechanical and fatigue properties, a higher Young’s modulus, and better abrasion resistance. They show increased impact energy absorption. This allows for a reduction in the number of fiber reinforcement layers, resulting in less thickness and weight. Introduction of conductive nanoparticles, such as carbon black, CNTs, graphite, graphene, or metals, gives composites the ability to conduct electricity. Due to these advantages, composites are used mainly in the arms industry, in the production of smart vests, helmets, and armor [23,25,26,27,28].

**Figure 3:**
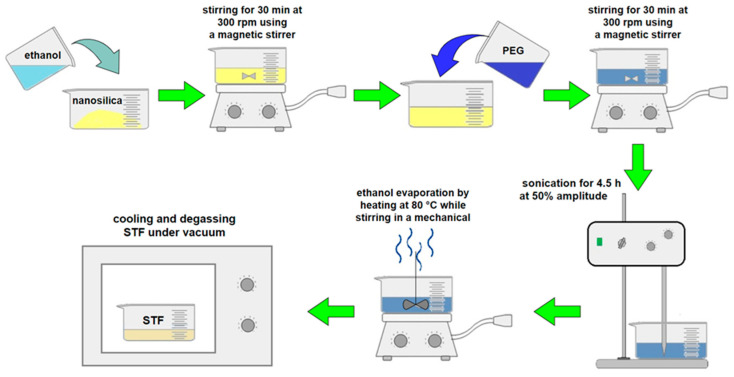
STF fabrication scheme based on the procedure in [34].

A composite is reinforced with nanoparticles by dispersion of the nanofiller in the matrix [23] or impregnation of the fibers or both techniques [26]. An interesting phenomenon of nanofiller growth on glass fibers was described by Nasser et al. They placed the fabric in zinc salt solution and coated the fabric fibers with a ZnO layer, which increased the stiffness and tensile strength and improved the adhesion of the fibers and the matrix as well as the energy absorption mechanism [29]. The following sections of this paper present the effects of the abovementioned methods on the mechanical strength of composites reinforced with glass, carbon, and aramid fibers. For composites dedicated to arms applications, fabrics impregnated with liquids (STF) and gels (STG) thicken in shear [27] or by the growth of nanofillers on them [30]. An STF is a non-Newtonian liquid consisting of two dispersion phases. The first phase is usually ethylene glycol (average molecular weight of 200, 400, or 600 g/mol) or propylene glycol (average molecular weight of 400 g/mol), in which silica with a particle size between 100 and 750 nm, calcium carbonate, or PMMA (the second phase) is usually dispersed (Figure 3) [3,31,32,33].

**Figure 4:**
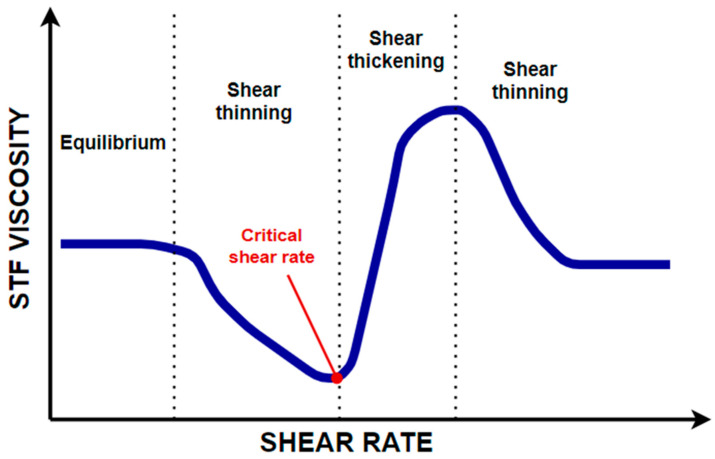
Overview graph showing the change in STF viscosity as a function of shear rate. [37]. (Adapted with permission from [37]. Copyright 2019 John Wiley and Sons).

The use of an STF increases the friction between the fabric of the fibers and energy absorption. This allows for a reduction in reinforcement layers, and thus, the thickness and weight of the composite, while maintaining the same strength. The use of larger SiO_2_ nanoparticles (about 500 nm) decreases the critical shear rate, improving the mechanism of action and efficiency of the STF. The critical shear rate is defined as the value of the shear rate at which a sharp increase in viscosity is observed (Figure 4). The STF changes from a liquid state to a nearly solid state [3,35]. The STG is a polymer that changes from a liquid state to a rubbery state when subjected to shear [36]. Similar to an STF, the use of an STG reduces the impact force by several tens of percentage points.

Additionally, an STF is more stable and insensitive to moisture. The hygroscopic nature of glycol in an STF makes it prone to absorbing moisture, which weakens the shear mechanism [27,36,38].

#### 2.1.1. Glass-Fiber-Reinforced Polymer Composites

Glass-fiber-reinforced polymer composites make up about 90% of all polymer fiber composites used in industry. Glass fibers in the form of roving, mats, fabrics, and chopped fibers are mainly used in the manufacture of boat hulls, yachts, tanks, bathtubs, roof gutters, pipes, and machine housings [5,39]. To improve the mechanical properties of epoxy-glass composites, Tate et al. separately introduced 6, 7, and 8 wt% of nanosilica, 20 nm in size, into the matrix. Improvements in mechanical properties were observed in all samples containing the filler (Table 2). The composite containing 6 wt% of nanosilica showed the highest increase in tensile strength (22%) and elongation and interlaminar shear strength (ILSS) (26%). The composite containing 7 wt% of nanosilica had the highest elastic modulus and flexural strength [40]. Ravi et al. investigated the effect of reinforcing the composite with PMMA and silicon carbide (SiC) beads. The addition of only PMMA (10 vol%) to the matrix increased the flexural strength and flexural modulus at the expense of elasticity compared to the composite reinforced only with glass fabric. The introduction of both PMMA beads (10 vol%) and SiC particles (1 vol%) increased the tensile and flexural strengths by 8% and 37%, respectively, compared to the fiber composite and 32% and 23%, respectively, compared to the sample containing PMMA [41]. Rahmat et al. prepared glass-fiber-reinforced composites with boron nitride nanotubes (BNNT). The addition of 1% BNNT improved the impact strength, flexural strength, and shear strength, on average, by 22%, 15%, and 8%, respectively [42]. Zeng et al. improved the mechanical properties of composites by grafting the glass fabric with multiwalled carbon nanotubes (MWCNTs). The fabrics were impregnated in a suspension of nanofillers in ammonium persulfate (APS) and ethanol solution. An increase of approximately 33% in flexural and tensile strengths was observed for the epoxy–glass composite containing carbon nanotubes compared to the reference sample. The Young’s modulus and flexural modulus increased by 41% and 36.7%, respectively, and the ILSS increased by 40.5%. The researchers also found that fabric impregnation eliminates the disadvantages that occur with dispersion in the matrix, i.e., the tendency to form agglomerates and the uneven dispersion of the nanofiller between layers and along fabrics in the infusion method. In addition, APS facilitated and affected the uniform saturation of the glass fabric and improved interfacial adhesion [43]. Vigneshwaran et al. investigated the mechanical properties of epoxy–glass composites upon the addition of 0.2, 0.6, and 1 wt% graphene nanoplatelets (GnP). One-half of the nanofiller was dispersed in the matrix, while the other half was used to coat the glass mat. Compared with the reference sample, the laminate containing 1 wt% GnP had twice the tensile strength and a 70% higher Young’s modulus. In addition, there was a 45% increase in impact strength and a 38% increase in energy absorption. The composite also exhibited 87% less surface damage area. Impregnation of the mat with GnP improved the adhesion of the fibers to the matrix [44].

Nasser et al. investigated the interfacial shear strength (IFSS) of epoxy composites reinforced with glass fibers coated with ZnO nanoparticles (NPs) and nanowires (NWs). The fibers were functionalized with an oxidizing mixture (sulfuric acid and perhydrol) to increase adhesion and enhance coverage. The wall strength at a quasi-static strain rate increased for ZnO NWs and NPs by 96% and 44%, respectively. At medium and high strain rates, IFSS saps of 29% and 68% were observed for ZnO NWs, respectively, and 27% and 22% for ZnO NPs, respectively. This result indicates the viscoelastic nature of the material, which can compensate for impact energy. This effect also reduces the probability of delamination or cracking of the reinforcement [29].

#### 2.1.2. Carbon-Fiber-Reinforced Polymer Composites

Carbon-fiber-reinforced polymer composites are extremely strong, lightweight, rigid structural materials resistant to high temperatures, friction, and corrosion. Because of these unique properties, they are used at a large scale in aviation, the automotive industry (machine skeletons and shells), armaments (ballistic shielding), and electronics (shielding enclosures) [25,50,51,52,53,54,55]. Tareq et al. investigated the effect of adding nanoclay and graphene to the carbon-fabric-reinforced composite matrix. Laminates containing 2 wt% nanoclay had the highest stiffness and the highest increase (28%) in the flexural modulus. Samples with graphite had the highest strength. The addition of 0.1 wt% of this filler resulted in a 21% increase in flexural strength. Compared with these samples, the composite containing both nanoadditives showed lower modulus and flexural strength. This was due to the dispersion time being too short [45]. Moghimi et al. also investigated the synergistic effect of reinforcing epoxy–carbon composites with two types of nanofillers. They used multiwalled carbon nanotubes (MWCNTs) and nanosilica in three ratios: 0.2%/0.7%, 0.7%/0.2%, and 0.45%/0.45% by weight. The sample containing equal amounts of both nanoadditives had the best mechanical and tribological properties. The tensile strength and Young’s modulus increased by 25.2% and 31%, respectively; the coefficient of friction decreased by 88%; and the wear resistance increased by 98%. SEM analysis showed good dispersion of nanofillers in the matrix, which improved interfacial adhesion [46]. Khan et al. carried out the functionalization of graphite nanoparticles in two ways: attachment of (3-glycidyloxypropyl) trimethoxysilane (GPTMS) and attachment of epichlorohydrin (EP). In addition to a reference sample, they fabricated graphite-reinforced epoxy–carbon composites: unmodified (N-CFRP), GPTMS-modified (G-CFRP), and EP (E-CFRP). The best mechanical properties were found for G-CFRP. The modulus and flexural strength increased by 34% and 36% for G-CFRP, by 16% and 16% for E-CFRP, and by 10% and 3% for N-CFRP, respectively. The tensile strength and Young’s modulus increased by 36% and 29% for G-CFRP and by 14% and 7% for N-CFRP, respectively. For E-CFRP, the tensile strength increased by 20% and Young’s modulus decreased by less than 10% [47]. Wang and Cai performed carbon fabric impregnation using a spray method (Table 3). The spray solution was a suspension of graphene nanoplatelets in an epoxy–acetone mixture. They prepared four laminates containing 0%, 0.1%, 0.3%, and 0.5% by weight of graphene. The uniform coating of the fabrics with the nanofiller increased the interfacial bonding and fracture toughness. The flexural modulus increased with the amount of filler. The sample containing 0.3% GnP showed the highest increase in flexural strength (27.2%) and the ILSS (24.5%) [56]. Badakhsh et al. performed a two-step carbon nanotube (CNT) impregnation of carbon fabrics. First, the cleaned fabrics were coated with nickel using electroplating. Then, CNTs were applied to the fabrics by gas phase chemical deposition. Nickel catalyzed the deposition and growth of CNTs. The highest efficiency was achieved at 15 wt% of nickel. In addition, the researchers developed a composite consisting of a carbon fabric coated with only a nickel layer and an epoxy resin in which CNTs were dispersed. For the composite reinforced with the Ni-CNT-modified carbon fabric, the flexural strength increased by 52.9% compared to the reference sample. The ductility index was 40% lower than that of the composite with dispersed CNTs [57]. Nasser et al. also deposited ZnO nanoparticles and wires on carbon fibers that were pre-functionalized with 70% nitric acid. The composites containing ZnO NWs showed a decrease of 62% and 73% in the IFSS, respectively, at medium and high strain rates; for ZnO NPs, the decrease was 40% and 58%, respectively. The results show an increase in the ballistic performance of composites reinforced with impregnated fibers [58]. Selver investigated the strength of epoxy–carbon and epoxy–glass composites reinforced with a shear thickening fluid. The STF was prepared by dispersing (10%, 15%, and 20% by weight) nanosilica in PEG. Glass- and carbon-fabric-reinforced composites containing 15 wt% of silica showed a 12% and 10% increase in tensile strength, respectively, and a 24% increase in Young’s modulus. Energy absorption also increased (up to 27%). However, the flexural strength of these composites deteriorated compared to the reference sample [59].

#### 2.1.3. p-Aramid-Fiber-Reinforced Composites

In the arms industry, p-aramid fibers, known commercially as Kevlar (DuPont) or Twaron (Teijin), are the main reinforcement of polymer composites used for helmets, bulletproof vests, body armor, and ballistic shields [4,19,62,63]. Suresha et al. reinforced epoxy–aramid composites by dispersing them in a matrix of 0.15, 0.3, and 0.5 wt% MWCNTs. The addition of the filler improved the interfacial adhesion. The sample containing 0.3 wt% MWCNTs had the best mechanical properties. The tensile strength and Young’s modulus increased by 46% and 22.1%, while the flexural strength and modulus increased by 74% and 54%, respectively. Additionally, impact strength improved (31.2%) [48]. Dharmavarapu and Reddy investigated the effect of adding (0.5%, 1%, and 2% by volume) modified nanosilica on the mechanical properties of epoxy–aramid composites. The silica was surface-treated with 3-aminopropyltrimethoxysilane (APTMS) by acid hydrolysis. The composite containing 1 vol% of nanofiller had the highest mechanical strength. The tensile strength, flexural strength, impact strength, and hardness increased by 27.5%, 17%, 67%, and 14%, respectively, compared to the reference sample. The addition of 1 vol% of modified nanosilica improved the energy absorption from 6.5 to 8.2 J [49]. APTMS can also be used to modify fibers. Jia et al. performed multistep grafting of 3-aminopropyltrimethoxysilane onto an aramid surface using γ-radiation, 1, 4-dichlorobutane, and sodium hydroxide. The modified fiber surface exhibited increased roughness. APTMS formed chemical bonds with the epoxy resin, resulting in improved interfacial properties. The IFSS of the laminate containing the modified aramid reinforcement increased by 51.03% compared to the reference sample [60]. Malakooti et al. subjected composites reinforced with aramid fabric impregnated with ZnO nanowires to ballistic and strength tests. The tensile strength and Young’s modulus of the composites increased by 13.2% and 8.8%, respectively, and the impact resistance increased by 66%. The presence of ZnO nanowires on the fiber surface increased the friction between the yarns and reduced their mobility in the fabric [30]. Aramid fibers show sensitivity to UV radiation. Zhang and Teng showed that after 168 h of UV exposure, epoxy composites reinforced with modified and impregnated aramid fibers showed 97.2% of the original tensile strength value. The fibers were functionalized with poly-L-3, 4-dihydroxyphenylalanine (PDOPA) and coated with ZnO. PDOPA facilitated the grafting and growth of ZnO nanowires and, as a whole, increased the surface roughness and improved the matrix–reinforcement adhesion [61]. Liu and Ávila studied the effect of the presence of an STF on composites reinforced with aramid fabrics. STF-reinforced composites based on silica and CaCO_3_ (75% and 25%, respectively, by weight) prepared by Ávila showed the best results in ballistic tests. The work required to stop bullets was 40% less compared to the reference sample. The researchers showed that the presence of an STF increased the friction between the yarns and led to deformation of the bullets. Additionally, the STF allowed the reduction of reinforcement layers from 32 to 19, while maintaining the same ballistic properties of the composite [64] (Table 4). The laminates made by Liu additionally reinforced with an STF based on silica and CNTs had more than 50% higher puncture resistance, absorbed 65% more energy, and thus, could withstand more impact force [65]. Dixit showed that STF impregnation increases energy absorption of the reinforced fabric by 10% more compared to pure Kevlar fabric. Additional coating of ZnO fibers increased the absorption by 36% compared to the control sample [66]. Zhao et al. focused on the impregnation of aramid reinforcement using an STG. They made 5-, 10-, 15-, and 20-layer laminates reinforced with an STG, and corresponding reference samples. In the ballistic tests, the impact force recorded by the detector decreased (from 805 to 223 N) as the layers of the STG-reinforced composite increased (from 5 to 20). For the reference samples, the impact force decreased from 1125 to 460 N. Additionally, composites containing an STG with carbon black absorbed 21.6% more impact energy. The addition of STG allowed for increased friction between the fibers, enabled the composites to absorb more energy, and improved their ballistic properties [28,38]. He et al. demonstrated synergies between STF and STG used to impregnate Kevlar fabric. Compared to composites impregnated with an STF alone, composites impregnated with a hybrid showed increased mechanical strength, elastic modulus, and impact resistance. Reducing fabric layers improved the energy dissipation mechanism and reduced weight and thickness. The addition of STG stabilized the protective coating of the STF, which increased the friction between fibers and their strength [37].

### 2.2. Hybridization of Fiber Reinforcement of Polymer Composites

**Figure 5:**
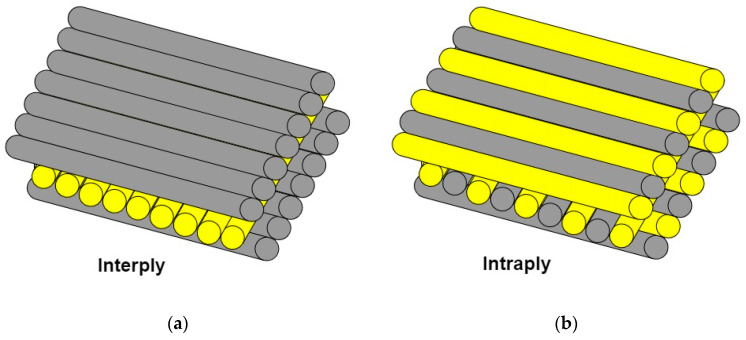
Hybrid fiber reinforcement systems of (**a**) interply and (**b**) intraply type.

Due to (fibrous) reinforcement, two types of hybrid composites are distinguished, layered (interply) and interwoven (intraply), which are shown in Figure 5.The first type, interply, consists of stacked layers of individual reinforcements in the form of fabrics or mats (Figure 5A).

In intraply, each reinforcement, in the form of a fabric or a mat, consists of several types of fibers, e.g., carbon and glass (Figure 5B) [67,68,69]. To obtain hybrid composites with the best possible mechanical strength, it is necessary to select appropriate fiber types. It is assumed that one is an expensive fiber with high Young’s modulus, and the other is a cheaper fiber with low Young’s modulus. The next step is to determine the order/sequence of their arrangement. The first layers are of a hard shear-resistant material that absorbs the impact energy. The middle and back layers should consist of tensile-resistant fibers. They accept and distribute the remaining energy. Usually, the top layers consist of fiberglass or p-aramid materials [70]. Randjbaran et al. showed that using glass fabric instead of Kevlar fabric in the first layer enables higher energy absorption [71]. The middle part is mostly carbon fiber reinforcement [72,73,74]. As the volume percentage of carbon reinforcement increases, the flexural and tensile strengths of the composite increase [75]. Carbon material is not recommended for use as the top layers. As mentioned earlier, glass reinforcement is preferred at the front, while Kevlar is used for the back layers [71,74,76].

#### 2.2.1. Influence of Ply Orientation on the Performance Properties of Hybrid Polymer Composites

The arrangement of reinforcement (fabrics) layers at different angles plays an important role in absorbing impact energy. In the case of unidirectional fabrics, during impact, the energy is distributed between the fibers along the 0° axis. When we apply another layer perpendicular to the first layer, the energy is distributed along the 0° and 90° axes. The trace after the impact resembles a quadrilateral pyramid. By adding more layers at different angles, the whole gain becomes increasingly isotropic.

**Figure 6:**
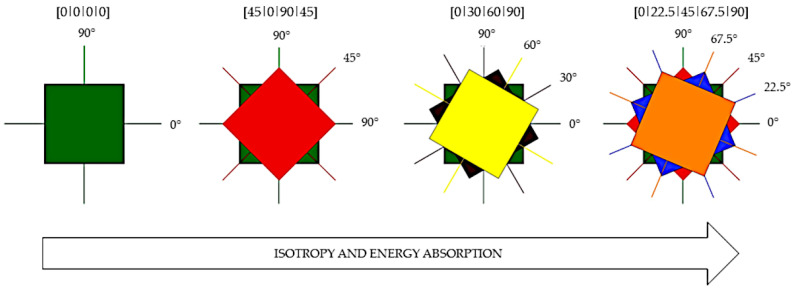
Angular orientations for the four reinforcement layers. (Adapted with permission from [78]. Copyright 2019 Elsevier).

The energy is distributed over increasing axes, and the post-impact shape is similar to a cone [3,77,78]. Figure 6 illustrates the four ways of orienting reinforcement layers. An increase is observed in the isotropy of the gain with the addition of another layer and changes in the orientation angle. The impact energy spreads in 10 directions for the [0/22.5/45/67.5/90] system and only in 4 directions for the [0/0/0/0] system. As previously mentioned, this translates into the composite’s ability to absorb energy. Researchers have shown that layer orientation allows the absorption of approximately 11% to 20% more impact energy. When orienting layers, it is recommended to keep the angles between the axes equal and as large as possible. For composites consisting of two, three, or four layers of woven cloth, the angles (0°, 45°), (0°, 30°, 60°), and (0°, 22.5°, 45°, 67.5°) are used sequentially. In subsequent layers, the analogy is followed or the given sequence is repeated several times [4,78,79,80,81].

#### 2.2.2. Effect of 2D and 3D Structure on Mechanical Properties of Hybrid Polymer Composites

Two-dimensional fabrics are commonly used as reinforcement for composites. Their properties depend not only on the fiber from which they are made but also on the weave. There are three basic weaves: linen, twill, and satin. Linen is characterized by symmetry, durability, and a tendency to fold. Threads of weft and warp pass alternately above and below each other. Twill is strong, smooth, and well-shaped. Each weft thread passes under and over the warp thread(s), creating a characteristic twill pattern. In satin weave, the warp threads are raised above the weft threads, providing the fabric with a smooth surface and easy draping ability 0 [4,58,82]. Among the aforementioned types, twill exhibits higher flexural, tensile, and shear strengths [80,83,84]. Cavallaro showed that the use and proper arrangement of fabrics with different weaves (linen and twill as the outer layer and satin as the inner layer) can increase their resistance and energy absorption capacity compared to laminates containing fabrics of one type [85].

In contrast to 2D fabrics, three-dimensional (3D) fabrics have an additional thread (binding) in the z direction, which is the thickness. They are characterized by stiffness and strength in x, y, and z directions; better structural integrity; and stress transfer between layers. Compared to 2D-reinforced composites, 3D-reinforced composites exhibit higher impact strength, flexural strength, compressive strength, and interlaminar fracture. Upon impact, they absorb and dissipate twice as much energy. Unlike 2D fabrics, the damage area is small and delamination is practically absent [3,83,86,87]. Among 3D fabrics, several types of structures are distinguished, of which orthogonal ones are the most popular and most commonly used as reinforcement in composites with increased mechanical strength. Due to their simple microstructure, high stiffness, strength in all directions, low cost, and efficient production, 3D fabrics are readily used in the aviation, automotive, and, especially, arms industries, where 2D/3D fabric hybrids are used as reinforcement for bulletproof vests or body armor [58,82,88].

## 3. Conclusions

Fiber-reinforced polymer composites are being increasingly used in the defense industry. However, for protective applications, in addition to high specific strength and stiffness, polymer composites are also required to have a high energy absorption capacity. Furthermore, the properties of polymeric composite materials are still clearly different from those of metallic and ceramic materials used in industry. Therefore, the first and most important stage of designing composite materials for the arms industry is appropriate selection of the matrix and reinforcement to obtain the required mechanism of action. The literature review presented in this article on improvements in the properties of polymeric composites provides information about their application in the arms industry. The selection of specific fillers and other modifiers and their introduction into polymer composites make it possible to change their properties with respect to the predicted working conditions. In addition to nanofillers, various types of fibers (glass, carbon, and aramid) and the fabrics obtained from them, with different weaves, orientations, and impregnation (STF and STG), are used in polymer composites. Their use mainly improves the composites’ strength against mechanical damage by enhancing energy absorption, thus reducing the area of damage. The multiple ways to improve the mechanical strength of composites and the possibility of their simultaneous use give scientists a wide spectrum of research, as well as an opportunity to develop new types of hybrid composites with unique properties. However, the replacement of metal alloys and ceramics by polymeric composite materials, to ensure economy of production, usually requires a complete change in the concept of product design. Therefore, a very significant challenge associated with hybrid composites is their technological and application capabilities. In addition, weight and thickness should be considered when hybrid fiber composites are designed. Evaluation of the literature data showed that research on impact-resistant polymer composites should be focused on the development of hybrid systems, i.e., combining matrix modifications (via STF) and an appropriate fiber reinforcement structure. Among others, this ability to modify makes them unique materials for the 21st century.

## Figures and Tables

**Table 1:** Summary of the mechanical properties of selected high-strength fibers [4,5,6,7,8].

Fiber	Density (g/cm^3^)	Tensile Strength (GPa)	Young’s Modulus (GPa)	Elongation at Break (%)
E glass fiber	2.63	3.5	68.5	4.0
S glass fiber	2.48	4.4	90.0	5.7
Carbon fiber(Celton)	1.80	4.0	230.0	1.8
p-Aramid (Kevlar 149)	1.47	3.5	179.0	1.6
m-Aramid (Nomex)	1.40	0.7	17.0	22.0
UHMWPE (Dyneema SK76)	0.97	3.6	116.0	3.8
Zylon AS	1.54	5.8	180.0	3.5
Zylon HM	1.56	5.8	270.0	2.5
Vectran	1.47	3.2	91.0	3.0
M5	1.70	5.8	310.0	1.4
Boron fiber	2.64	3.5–4.2	420.0–450.0	3.7
Silicon carbide	2.80	4.0	420.0	0.6
Alumina III (Nextel)	2.50	1.7	152.0	2.0

**Table 2:** Summary of publications in which the epoxy matrix was modified with nanofillers.

Ref.	Reinforcement Fiber Type	Filler	Content	Effect
[40] Tate et al.	Glass fiber	SiO_2_	6, 7, and 8 wt%	Increase in tensile, flexural, and interlaminar shear strengths Increase in modulus and elongation
[41] Ravi et al.		PMMA	10 vol%	Increase in tensile strength, flexural strength, and modulus Improved thermal stability and abrasion resistance
		SiC	1 vol%	
[42] Rahmat et al.		BNNT	1 wt%	Increase in flexural, shear, and impact strengths
[44] Vigneshwaran et al.		GnP	0.2, 0.6, and 1 wt% (of which 50% was used to impregnate the fiber)	Increase in impact energy absorption, tensile strength, and modulus Reduction in surface damage area Improved adhesion between components
[45] Tareq et al.	Carbon fiber	Nanoclay	2 wt%	Increase in flexural strength and modulus when added separately Higher stiffness and GnP with the best thermomechanical stability in samples with nanoclay
		GnP	0.1 wt%	
[46] Moghimi et al.		MWCNTSiO_2_	0.2 and.7 wt%	Increase in tensile strength and Young’s modulus Reduction in the abrasion coefficient Improved interfacial adhesion
			0.7 and 0.2 wt%	
			0.45 and 0.45 wt%	
[47] Khan et al.		N-CFRP		Improvement of tensile and flexural strength and modulus by modified graphite
		G-CFRP		
		E-CFRP		
[48] Suresha et al.	Aramid fiber	MWCNT	0.15, 0.3, and 0.5 wt%	Increase in tensile strength, flexural strength, modulus, hardness, and impact strength
[49] Dharmavarapu and Reddy		SiO_2_ modified with APTMS	0.5, 1, and 2 vol%	Improved tensile strength, flexural strength, impact strength, and hardness Increase in impact energy absorption

**Table 3:** Summary of publications in which the reinforcement was modified with nanofillers.

Ref.	Reinforcement Fiber Type	Filler/Impregnator	Effect
[43] Zeng et al.	Glass fiber	MWCNTs modified with APS	Increase in tensile strength, flexural strength, and modulus Improved ILSS and interfacial adhesion
[29] Nasser et al.		ZnO nanoparticles functionalized by piranha solution	Decrease in IFSS at medium and high and increase at low strain rates Improved interfacial adhesion
		ZnO nanowires functionalized by piranha solution	
[56] Wang and Cai	Carbon fiber	GnP	Increase in flexural strength, interlaminar shear, flexural modulus, and thermal conductivity
[57] Badakhsh et al.		Nickel (galvanization): phase I CNT (gas-phase deposition): phase II	Improved flexural strength Decrease in electrical resistance and ductility index
[58] Nasser et al.		ZnO nanoparticles functionalized by 70% nitric acid	Decrease in IFSS at medium and high strain rates
		ZnO nanowires functionalized by 70% nitric acid	
[60] Jia et al.	Aramid fiber	Grafting of APS by γ-ray and chemical treatment	Increase in fiber surface roughness and IFSS
[30] Malakooti et al.		ZnO nanowires	Increase in Young’s modulus, tensile strength, and impact strength
[61] Zhang and Teng		PDOPA functionalization and ZnO nanowire coating	Increase in UV resistance fiber surface roughness Improved IFSS and interfacial adhesion

**Table 4:** Summary of publications that used reinforcement impregnated with an STF/STG.

Ref.	Reinforcement Fiber Type	Impregnation Type	Filler in STF/STG	Filler Content	Effect
[59] Selver	Glass fiber	STF	SiO_2_	10, 15, and 20 wt%	Improvement in tensile strength, Young’s modulus, and energy absorption for 10 and 15 wt% Decrease in flexural strength and modulus for all samples
	Carbon fiber				
[64] Ávila et al.	Aramid fiber		SiO_2_	0, 25, 50, 75, and 100 wt% of the filler mixture	Increase in the impact energy absorption and friction between fibers
			CaCO_3_	0, 25, 50, 75, and 100 wt% of the filler mixture	
[65] Liu et al.			CNT		Increase in resistance to fiber pull-out strength and puncture Increase in energy absorption
			SiO_2_	71 wt%	
[66] Dixit et al.			SiO_2_	65 wt%	Increase in fiber pull-out strength and impact energy absorption
			Impregnation of ZnO nanowires		
[38] Zhao et al.		STG			Increase in impact energy absorption and friction between fibers
[28] Zhao et al.			Carbon black		Increase in impact energy absorption Mechanical–electrical coupling in the form of a change in resistivity as a function of impact energy, due to addition of carbon black
[36] He et al.		STF + STG	SiO_2_ (STF)		Increase in impact strength and modulus Improved energy dissipation mechanism Reduction in composite weight and thickness STF stabilization and increase in traction between fibers, due to addition of STG

## Data Availability

Not applicable.
